# Cerebral Venous Sinus Thrombosis in a Child with Idiopathic Nephrotic Syndrome: a case report

**DOI:** 10.1590/2175-8239-JBN-2018-0009

**Published:** 2018-08-02

**Authors:** Ana Isabel Rodrigues Silva, João Tavares, Ana Sofia Vaz, Nádia Brito, Mónica Vasconcelos, Teresa Sevivas, Lurdes Moura, Carolina Cordinhã

**Affiliations:** 1 Centro Hospitalar e Universitário de Coimbra, Hospital Pediátrico, Coimbra, Portugal.; 2 Hospital Distrital da Figueira da Foz, Serviço de Pediatria, Coimbra, Portugal.; 3 Centro Hospitalar e Universitário de Coimbra, Centro de Desenvolvimento da Criança, Hospital Pediátrico, Neuropediatria, Coimbra, Portugal.; 4 Centro Hospitalar e Universitário de Coimbra, Serviço de Sangue e Medicina Transfusional, Coimbra, Portugal.; 5 Centro Hospitalar e Universitário de Coimbra, Hospital Pediátrico, Unidade de Nefrologia Pediátrica, Coimbra, Portugal.

**Keywords:** Nephrotic Syndrome, Child, Intracranial Thrombosis, Síndrome Nefrótica, Criança, Trombose Intracraniana

## Abstract

Complications are rare in pediatric cases of idiopathic nephrotic syndrome (NS). Thromboembolism ranks among the most uncommon and difficult complications to diagnose, particularly in the first episode of NS, since clinical signs might be unspecific. This report describes the case of a 5-year-old girl with NS for the first time presenting with severe hypoalbuminemia (< 2g/dL). The patient responded poorly to therapy with corticosteroids. On day 8 of hospitalization she started having headaches and vomiting; she did not present hemodynamic alterations, fever or exanthems, and her neurological parameters were normal. The patient was suspected for intracranial hypertension, and computed tomography scans revealed she had cerebral venous sinus thrombosis (CVST). She was started on anticoagulants and showed clinical signs of improvement. The patient had no evident prothrombotic risk factors. She had three other episodes since she was diagnosed, one in which her plasma antithrombin level was low. Although antithrombin levels were normal in her first episode, she was tested after the resolution of proteinuria. The low levels of antithrombin seen in the first recurrence might have mirrored the initial drop in plasma antithrombin levels, an idea supported by the severe hypoalbuminemia she had when diagnosed. This severe manifestation of acquired thrombophilia might be in the origin of CVST. This report presents a rare case of thromboembolic complication in a pediatric patient with NS. The patient progressed well since she was started on anticoagulants. Although she did not present any evident risk factors at first, the development of her case indicated that severe acquired thrombophilia might have worked as the pathophysiological mechanism leading to CVST.

## RESUMO

A Síndrome Nefrótica (SN) idiopática em crianças pode, raramente, complicar-se. O tromboembolismo é uma das complicações mais raras, principalmente no primeiro episódio, e de diagnóstico mais difícil, uma vez que a clínica pode ser inespecífica. Descrevemos o caso de uma criança de 5 anos com episódio inaugural de SN, destacando-se hipoalbuminemia inicial grave (< 2g/dL). Apresentou fraca resposta inicial à corticoterapia e, após 8 dias de internamento, iniciou quadro de cefaleias e vômitos, sem alterações hemodinâmicas, sem febre, sem exantema e com exame neurológico normal. Perante a suspeita de hipertensão intracraniana, foi realizada TC-CE, que mostrou trombose venosa cerebral (TVC). Foi então iniciada terapêutica anticoagulante com posterior boa evolução clínica. Trata-se de uma criança sem fatores de risco pró-trombóticos evidentes. Desde o diagnóstico, teve 3 recaídas, uma das quais com níveis baixos de antitrombina, que no episódio inaugural eram normais, apesar de avaliados já numa fase não proteinúrica. Suspeita-se, assim, que esse déficit plasmático em antitrombina na recaída poderá mimetizar a queda plasmática inicial, hipótese também apoiada pela hipoalbuminemia grave ao diagnóstico. Esta trombofília grave adquirida poderá ter sido mecanismo etiológico para a trombose venosa cerebral. O interesse deste caso prende-se com a raridade de complicações tromboembólicas na SN Pediátrica, ainda mais raras no episódio inaugural. Nesse caso, a boa evolução foi possível após a associação da terapêutica anticoagulante. Embora sem fatores de risco iniciais evidentes, a evolução do caso permitiu a suspeita de uma trombofília adquirida grave como mecanismo fisiopatológico do tromboembolismo cerebral.

## INTRODUCTION

Nephrotic syndrome (NS) is the most frequent glomerular disease in childhood, affecting 2-7:100000 children.^1^ Minimal change disease accounts for 95% of pediatric cases, usually with good prognosis.^2^

Infection, anasarca, hypovolemic shock, anemia, renal failure, hormonal alterations, and idiosyncratic reactions rank among the most common complications. Thromboembolism is a rare event, and cases of cerebral venous sinus thrombosis (CVST) have been sparsely reported in the literature.^3^

Thromboembolism occurs in 0.6% to 3% of the cases of NS, particularly in individuals with focal segmental glomerulosclerosis or severe and/or prolonged proteinuria.^4^ Clinical manifestations vary. Patients may be asymptomatic, a factor that may cause the disease to be underdiagnosed.^1^ The most frequently involved vascular territories are the pulmonary artery, the renal vein, the inferior vena cava, and the femoral vessels. The risk factors described in the few case reports on CVST include severe proteinuria, steroid resistance, and having a central venous catheter.^5^

The pathophysiology of thromboembolism in the context of NS is multifactorial and relates to the urinary excretion of antithrombotic factors (particularly antithrombin [AT]), procoagulant activity and increased platelet aggregation, and thrombocytosis, all of which contribute to the installation of a state of hypercoagulability in affected individuals.^6^ When used alone, diuretics increase the risk of dehydration and function as a predisposing factor.^7^

The clinical manifestations of CVST in children are unspecific, but the presence of neurological symptoms in contexts of relapsing NS invokes the complication. CVST is even rarer in patients having their first episode of NS. Clinical suspicion plays a pivotal role in early imaging assessment and prompt prescription of anticoagulant therapy, all of which factors connected to better prognosis.^8^

## CASE REPORT

A previously healthy five-year-old girl was taken to the emergency unit on account of periorbital and tibial edema developing for three weeks. Edema worsened in the morning and improved throughout the day. She had had productive cough for five days without fever or other symptoms.

Her maternal grandfather had thrombophilia (unknown type) and was on chronic anticoagulant therapy.

Initial examination revealed she had normal blood pressure, periorbital and tibial edema, and that her body weight had increased by 20% since the last time she had been weighed seven months prior. The main findings derived from her tests were nephrotic proteinuria (urine protein to creatinine ratio: 6.9 mg/mg), hypoalbuminemia, and hypercholesterolemia (Table 1). She was initially diagnosed with NS and prescribed corticosteroids (prednisolone 60 mg/m^2^/day). Her respiratory condition deteriorated within the first three days of hospitalization; she was afebrile and had persisting proteinuria and edema. On day 3 of hospitalization she was started on amoxicillin (80 mg/kg/day), and on day 5 of corticosteroid therapy the edema regressed and her body weight decreased by 1.5 kg (7%); her condition was stable until day 7. On day 8 she started waking up with headaches in the middle of the night associated to morning vomiting. She was hemodynamically stable, her blood pressure was within normal range, and she did not have exanthems, meningeal or focal neurological signs.

**Table 1:** Initial analytical assessment

Hemoglobin (g/dL)	14.5	pCr (mg/dL)	< 0.1
Leukocytes (/uL)	20300	Total cholesterol (mg/dL)	558 (↑)
Platelets (/uL)	689000 (↑)	Albumin (g/dL)	1.6 (↓)
Creatinine (mg/dL)	0.5 (N)	Total Proteins (g/dL)	4.2 (↓)
Urea (mg/dL)	6.2 (N)	C3/C4 (mg/dL)	156/27
Na (mEq/L)	131	IgG (mg/dL)	180 (↓)
K (mEq/L)	4.3	Proteins/ creatinine (urine) (mg/mg)	6.9 (↑)
Cl (mEq/L)	99	Proteinuria (mg/m^2^/h)	76.4 (↑)

**Figura 1:**
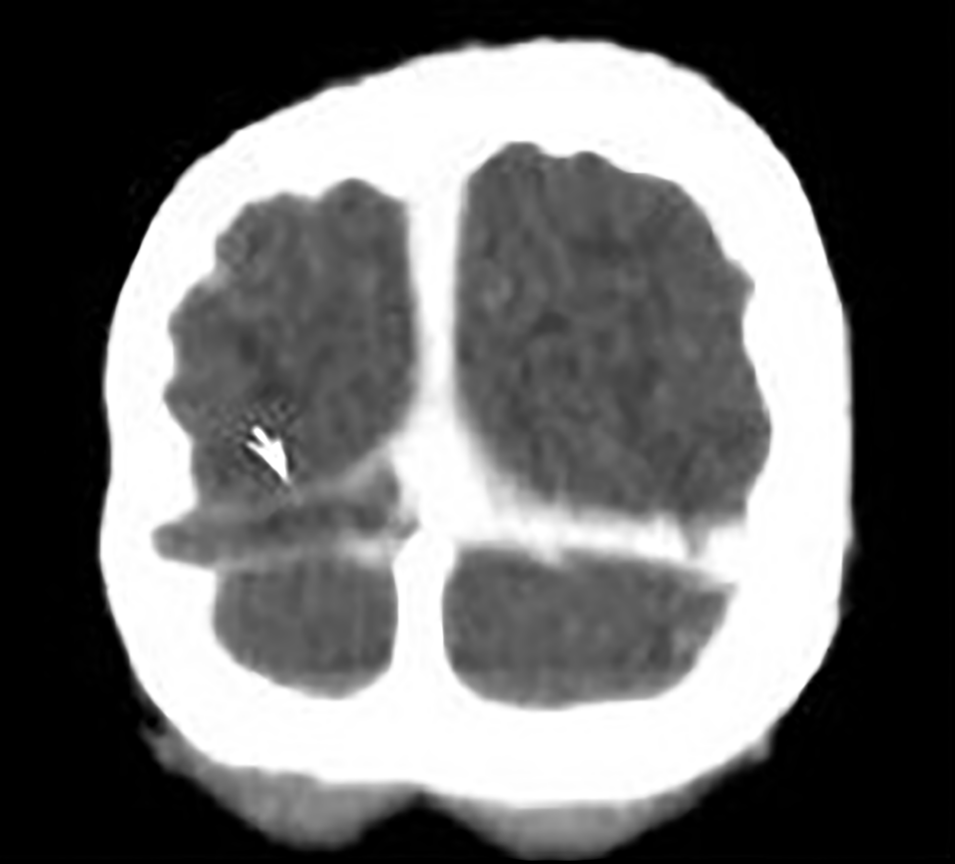
Baseline head CT scan (Electronic Patient Chart).

**Figura 2:**
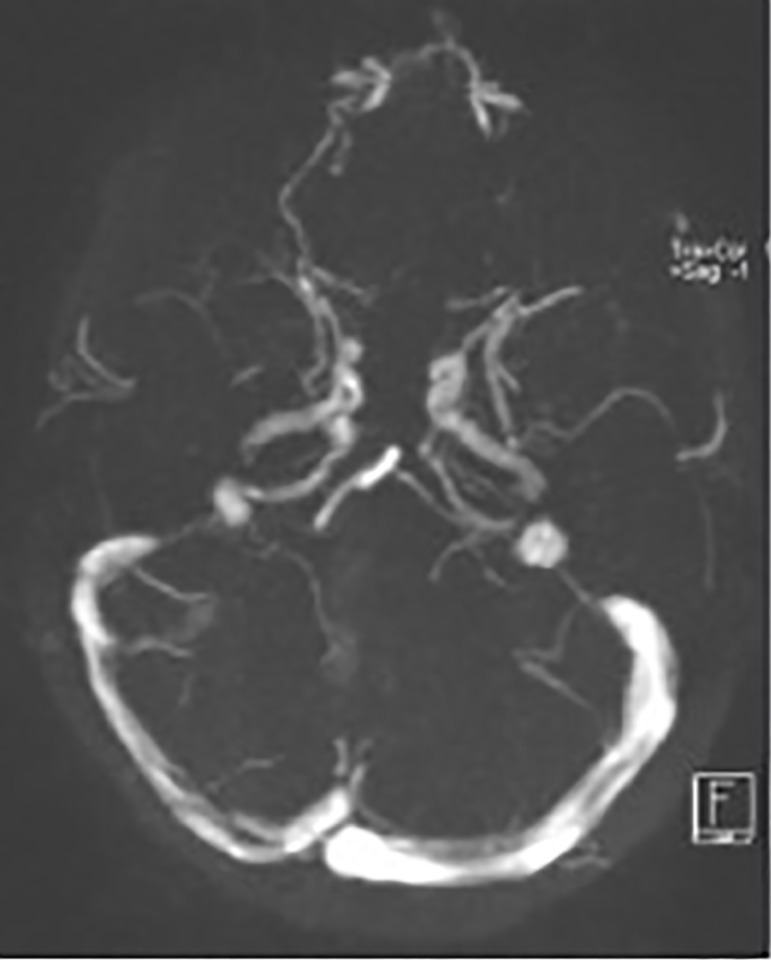
Baseline head MRI. Source: Electronic Patient Chart.

Computed tomography (CT) scans showed "hyperdense lateral sinuses and torcular herophili" (Figure 1). Additional contrast-enhanced CT scans confirmed filling defects in the right transverse sinus when compared to the contralateral sinus. The patient was thus diagnosed with right transverse sinus thrombosis. She was referred to a tertiary hospital (Figure 2) and was started on subcutaneous enoxaparin followed by warfarin (target INR 2-3). She was kept on corticosteroids.

Her headaches gradually improved and she became asymptomatic after two days of antithrombotic therapy. On day 13 of hospitalization, she no longer had proteinuria. The patient was discharged with a prescription of warfarin and corticosteroids after spending 23 days in hospital.

Tests for prothrombotic factors (performed after she had been on prednisolone for eight days and one day without proteinuria) did not show alterations: protein C 180% (N > 51%), protein S 88% (N > 53%), homocysteine 4 umol/L (N > 0.88 umol/L), lupus anticoagulant test - negative, antithrombin 149% (N > 76%), normal Leiden factor V, PRT 20210GA, and MTHFR677CT; her tests were negative for antinuclear and cytoplasmic antibodies, anti-SSA60, SSB, Sm, RNP, Scl70, JO1, cardiolipin, IgG, and IgM antibodies.

A multidisciplinary team covering the areas of pediatric nephrology, neurology, and hemostasis followed the patient. Control head MRI scans taken three months later showed the vessel was patent again, and the patient was started on corticosteroid therapy (6 weeks of daily prednisolone 60mg/m^2^/day followed by 6 weeks at a dosage of 40mg/m^2^/day in alternate days). She was taken off anticoagulants ten months after the initial event.

The patient had three other episodes of NS in contexts of viral upper airway infections seven, ten, and eleven months later. In one of the episodes she had low plasma antithrombin levels. She did not experience complications in the form of thromboembolic events.

## DISCUSSION

This report describes a case of idiopathic pediatric NS complicated by transverse sinus venous thrombosis *ab initio*. In this case, the interest lies in the rarity of thromboembolic complications in pediatric NS and the even rarer occurrence of CVST in a patient without identifiable prothrombotic risk factors. Diagnostic efforts are of particular importance in cases like this.

When present, thromboembolic complications are more frequent in relapsing NS.^3^ The surprise in the reported case was that the complications manifested a few days after the patient had been diagnosed, although she had had edema for three weeks and showed no evident risk factors.

CVST may have been triggered by acquired thrombophilia secondary to massive AT urinary excretion. The molecular weight of AT is lower than the molecular weight of albumin; therefore, decreases in its plasma levels caused by proteinuria are usually proportional to hypoalbuminemia. In the reported case, the baseline plasma AT level was normal, but the patient was tested after the resolution of proteinuria. The cessation of urinary protein excretion is one of the first factors to help restore AT levels.^9^ Her initially severe hypoalbuminemia (< 2g/dL) served as indication that her AT levels were low; the observation of decreased AT levels in relapsing NS suggested that the initial mechanism might have been mimicked.

Infection may deteriorate prothrombotic states and might have contributed to the onset of CVST. In a series with 17 cases of pediatric NS complicated by thromboembolism, two patients had infection as the sole prothrombotic risk factor.^10^

The clinical signs of CVST are age-dependent. Our patient presented with headaches and signs of intracranial hypertension. Only focal signs might be present, such as motor impairment and visual alterations.^5^ Analytical examination and early imaging play a key role in diagnosis, since good progression relies on the prompt introduction of adequate therapy and antithrombotic agents.

Head computed tomography is the most frequently used imaging test, with the cord sign pathognomonic of cortical vein thrombosis seen as a linear or curved hyperdense sign on the cerebral cortex. Head CT has its limitations, since in up to 40% of the cases it yields false-negative results, which become more probable when images are produced in later stages of involvement. Head magnetic resonance imaging is useful to characterize the lesions. Lumbar puncture is not routinely recommended.^11^

The most frequent sites of CVST in the pediatric population are the superior sagittal sinus and the transverse sinus, as in the reported case. The sigmoid sinus is affected in rare cases.^12^

Although the prothrombotic state of NS per se justifies the occurrence of thromboembolism, the rarity of this finding in pediatric populations requires that patients be screened for hereditary thrombophilia.^13^

In addition to corticosteroids to induce the remission of NS and manage proteinuria, therapy also includes antithrombotic agents.^14^ After the diagnosis of thromboembolic complication has been confirmed, it is recommended that antithrombotic therapy be started immediately with an initial scheme using low molecular weight heparin for five to seven days followed by oral administration for another three to six months (target INR 2-3). Therapy must be maintained until remission or for as long as nephrotic-range proteinuria and/or hypoalbuminemia < 2g/dL are present. This therapy has no proven prophylactic effect in cases of NS without thromboembolism.^15^

Thirteen percent of the individuals with baseline Glasgow scores below 12 - a marker of poor prognosis - have relapsing NS within 12 to 18 months of the first episode. Other factors associated with relapsing NS are hypoalbuminemia < 2g/dL - as seen in our patient - for less than two years, fibrinogen > 6g/L, AT < 70%, non-rerouting the vessel, and congenital thrombophilia, namely prothrombin G20210A of factor II mutation.^14^

Long-term morbidity is mainly linked to neurological sequelae in the form of motor impairment, seizures, and delayed psychomotor development. The sequelae from CVST in neonates may manifest only when the child reaches school age, in the form of attention deficit and learning disorders.^3^^,^ 8

CVST in pediatric NS is a rare condition possibly associated with permanent neurological sequelae. The rarity of this finding and its unspecific clinical signs make it a difficult-to-diagnose condition. Therefore, clinical suspicion is a very important tool in the diagnosis of CVST. Early imaging is needed to confirm the diagnosis. Good clinical evolution relies on the introduction of proper therapy with antithrombotic agents.
